# Assessment of knowledge, accessibility and utilization of palliative care services among adult cancer patients at Tikur Anbesa Specialized Hospital, Addis Ababa, Ethiopia, 2014: a cross-sectional institution based study

**DOI:** 10.1186/s13104-015-1630-x

**Published:** 2015-11-07

**Authors:** Serawit Lakew, Hasna Musema, Tsehay Shimeles, Julia Challinor

**Affiliations:** Department of Nursing and Midwifery, Arba Minch College of Health Sciences, Arba Minch, South West Ethiopia; Department of Oncology, Tikur Anbesa Specialized Hospital, Addis Ababa University, Addis Ababa, Ethiopia; Department of Nursing and Midwifery, Addis Ababa University, Addis Ababa, Ethiopia; School of Nursing, University of California, San Francisco, USA

**Keywords:** Palliative care, Adult cancer, Tikur Anbesa Specialized Hospital, Addis Ababa, Ethiopia

## Abstract

**Background:**

Cancer has been the leading cause of death worldwide for more than two decades. More than 150,000 cancer cases were estimated to exist in Ethiopia each year. The goal of cancer palliative care (PC) services are to prevent and relieve suffering and to support the best possible quality of life (QOL) for patients and their families, regardless of the stage of disease or the need for other therapies. This study attempted to assess the knowledge, accessibility and Utilization of PC Services for adult cancer patients by their perspective at Tikur Anbesa Specialized Hospital (TASH), Addis Ababa, Ethiopia.

**Method:**

A cross-sectional Institution based study was conducted among respondents who had Cancer at TASH. TASH was the only referral center of PC and cancer treatment in Ethiopia. The Hospital was selected for this study purposively. Data was collected by interviewing the client’s using a pretested Amharic version questionnaire. During the survey, 384 respondents with cancer were interviewed. Data entry was done using Epi Info version 3.5.2 and exported to SPSS version 20 for analysis. Logistic regression model was applied to control confounders.

**Result:**

Of the total clients interviewed, more than 62.2 % respondents had previous knowledge for cancer PC services. About 86 % of client’s were in the age 35 years and older. About 9 out of 10 (89.8 %) respondents reported problems on accessibility of PC services. Respondents previous knowledge of PC services (AOR = 26.9, 95 % CI 12.3, 59), presence of little (more than 75 % of physical problems/symptoms responded) physical well being of the respondents (AOR = 3.1, 95 % CI 1.96, 4.9), full (all social relationship problems responded as good/positive by respondents) social well being of the respondents (AOR = 1.7, 95 % CI 1.01, 2.8); monthly income $US 25–50 of the respondents (AOR = 0.25, 95 % CI 0.09, 0.7) and marital status single (never married) (AOR = 55.4, 95 % CI 1.2, 2660.4) were significantly associated with respondents utilization of PC services.

**Conclusion:**

High number of respondents reported problems on accessibility of PC services for cancer in TASH and more than just an average of respondents REPORTED presence of previous knowledge. Respondent’s previous knowledge about services, physical well being, social well being, income and marital status were a concern for utilization of cancer PC services at TASH. Health care providers at TASH will be recommended to have a sustainable health education session program on cancer PC services to adult clients after diagnosis of cancer.

## Background

Cancer has been the leading cause of death worldwide for more than two decades. In Africa, it is an emerging public health issue, with estimated 715,000 new cases and 542,000 deaths in 2008 only [1, 2]. Roughly, half a million people die of cancer in sub-Saharan Africa every year [3]. The FMOH (Ethiopia) estimated that there could be more than 150,000 cancer cases in Ethiopia each year though available data was limited [1, 4]. About 2013 adult cancer patients visited the Tikur Anbesa Specialized Hospital (TASH) in 2012 with in Addis Ababa city administration [4]. Comprehensive cancer registration and population-based measurement of cancer burden are yet to be done in Ethiopia [1].

World Health Organization (WHO) defined palliative care (PC) as an approach that improves the quality of life (QOL) of patients and their families facing the problem associated with life-threatening illness, through the prevention and relief of suffering by means of early identification and impeccable assessment and treatment of physical symptoms; psychological symptoms; social needs that include interpersonal relationships, caregiving, and economic concerns; and spiritual needs [5–8]. Its goal is to prevent and relieve suffering and to support the best possible QOL for patients and their families, regardless of the stage of the disease or the need for other therapies [5, 9, 10]. So, PC services are appropriate and should be available for all patients from the time of diagnosis with a life-threatening or debilitating conditions simultaneously with standard chemotherapy [9, 10]. But, most African PC researches and services were focused on patients with HIV/AIDS rather than patients with cancer [11].

PC has become an important part of the continuum of care for cancer patients. Many studies supported these as QOL [7, 12, 13, 14, 15, 16]. Services of PC must be individually integrated into specific care settings (such as, hospital, nursing home, assisted living, and/or home care) [9]. Survivors may experience a wide range of side effects that persisted for a long period. These side effects can reduce cancer survivors’ QOL [17]. Lack of public awareness about early detection, treatment of invasive cancer and PC services are barriers in countries with limited cancer treatment services (such as, Ethiopia) [18].

Patients with cancer could encounter from pain and weight loss up to anxiety and confusion as physical symptoms [19]. These symptoms often have a major impact on patients’ QOL. Good control of these symptoms are one of the most important aspects of care to patients and requires comprehensive interdisciplinary services [20]. These symptoms are infrequently treated by conventional care. So, PC programs have been developed to fill this gap in client care [21].

In India, PC policy established for the first time to facilitate the community-based home care initiatives under the leadership of local self-governments (LSGs) of Kerala state [22]. This service was only available in a few towns and three hospitals with the use of morphine in Tanzania. There is still no formal training program in Tanzania for any discipline of oncology. Clients, therefore, forced to travel to the abroad for the services and treatment need [23].

Studies outside home observed that 60.6 % clients with cancer had received medical care, which included drugs for their symptoms and specific diseases. 69 (66.3 %) had received some forms of supportive services like cash or kinds. Four had received water beds, two received walkers, and one received wheel chair. 31 (29.8 %) received catheter care which included putting, changing, or bladder wash during this period. Seven (6.7 %) were receiving ulcer care and two (1.9 %) were receiving infection care [24].

Nigerian study showed that 66 % of cancer patients were given a charity home by a philanthropic group to accommodate patients referred from far distance. All the patients were glad to have been introduced to PC service of the hospital. About 83 (46.6 %) clients however regretted non availability of similar service as home based for continuum of care. At the time of this review, about 65 (36.5 %) had gone back to their respective home base from where they were referred with symptoms well controlled, 102 (57.3 %) reported dead, and 11 (6.2 %) were still in PC services. Majority of patients (48.88 %) with PC services were in 41–60 age bracket [25].

As New York and other USA studies suggested, PC services were significantly associated with significantly lower likelihood of ICU use and lower in-patient costs compared to usual care [26]. These cost-related barriers to PC services are growing due to declines in coverage by employer-sponsored health insurances; increases in health insurance premiums, deductibles, and copayments; and rising costs of medical care in USA [27].

Kuwait study showed that cancer patients were significantly older than others (P < 0.0001). The patients were predominantly married (62.4 %), formally not employed (such as: housewives, 82.9 %), and only 31.5 % had up to high school education. Although the cancer patients were significantly more likely to be divorced or widowed (P < 0.001), there were no significant differences in occupation and education of patients [28]. In other study, older people are less likely to use PC by trained provider [OR ranged from 0.33 (0.15–0.72) to 0.82 (0.80–0.82)] [29]. Therefore, the objective of this study was to assess knowledge, utilization and accessibility of PC services by the perspectives of clients in TASH, Addis Ababa, Ethiopia.

## Methods

### Study design and setting

Hospital based cross sectional study was conducted from Feb 1 to May 1, 2014. TASH is located in Lideta sub city, Addis Ababa, Ethiopia. It was the only tertiary referral hospital in Ethiopia where cancer PC services and treatment virtually exists. The Hospital had 18 beds devoted to cancer care [4] and administered by Addis Ababa University. There were three in-patient and two out-patient rooms in oncology department.

### Sample size and sampling procedure

Sample size was determined by using single population proportion (SPP) formula based on the assumptions of 95 % confidence level, 50 % p value (no local study so far) and a 5 % non-response rate. Accordingly, the total sample size was 403 respondents with diagnosis of adult cancer. Systematic sampling technique was used to select the study subjects. Client’s registration number was used from the oncology unit. Around 2013 clients with adult cancer visited the Hospital in previous year (2012) and around 503 clients visited the unit for three consecutive months [30]. This, therefore, used for sampling patient flow estimation of the current study. Every respondent in the order of visiting the unit were included in the study until the required sample size achieved in the study period (since k = 1.2).

### Measurement

Data were collected using face-to-face client interview questionnaires. Knowledge of the client on PC services were asked as “any previous awareness of lists of palliative services.” Average and more responses were considered as knowledgeable and below average taken as not-knowledgeable. Variables for physical well being were used, such as lack of energy and nausea to feeling ill. Social well being responses, such as closeness to own friends, supported from them, closing to own partner, etc. were used. For each question there was corresponding scale of response (such as: no, little, somewhat and full).

### Statistical analysis

The data were coded, edited and entered into Epi-info version 3.5.2, cleaned and analyzed by SPSS for windows version 20. Frequencies, proportions and summary statistics were used to describe the study population in relationship to the relevant variables. Statistical analysis had three steps: first association was done between potential predictors of socio-demography, physical well-being, Social well-being and knowledge of the client’s for Utilization of PC services using bivariate analysis and 95 % confidence intervals to show existence of bivariate association. Next, to identify the independent contribution of each variable multivariate logistic regression model was used for the variables having association (p < 0.05) in bivariate logistic regression model. Finally, it was evaluated that variables identified as associated (p < 0.05) with the outcome variable in the multivariate analysis were used to predict the existence of association.

### Data quality control

Data Collection tool was adopted from previous studies outside the country and pretested [31]. Two days training were given to data collectors and supervisors. Every day completed questionnaires were reviewed and checked for completeness and relevance by the supervisors and Principal investigators. All the necessary feedback was offered to data collectors in the next morning before the actual procedure. Data checked for completeness, coded, entered into computer, cleaned and frequency checked for outliers and missing values before analysis.

### Ethical issues

Ethical clearance was obtained from Addis Ababa University, School of Allied Health Sciences Institutional Review Board (IRB). The study was commenced after letter of cooperation written to TASH from Federal Ministry of Health (FMOH), Ethiopia and Addis Ababa University School of Allied Health Sciences. Informed verbal consent was secured to each of study subjects. Each respondent was informed about the objective of the study and assurance of confidentiality, risks and benefits.

## Results

Most of the selected respondents participated in the survey (384 out of 403). The response rate was 95 %. Majority respondents 383 (89.3 %) were above the age 34 years with mean age of 45.8 years and ±11.3 years standard deviation. Most respondents were married 238 (62 %) and belong to Christian Orthodox 190 (49.5 %) religion. Median house hold income was $US 37.5 (Table 1).

**Table 1:** Socio-demographic characteristics of the respondents who had cancer, Tikur Anbesa Specialized Hospital, Addis Ababa, Ethiopia, 2014

Characteristics	Number	Percent
Age (years)		
<35	41	10.7
≥35	343	89.3
Mean ± SD^a^	45.8 ± 11.3	
Marital status		
Single	8	2.1
Married	238	62.0
Widowed	85	22.1
Separated	46	12.0
Divorced	7	1.8
Religion		
Christian orthodox	190	49.5
Christian protestant	71	18.5
Muslim	103	26.8
Others^b^	20	5.2
Ethnicity		
Tigray	44	11.5
Amhara	146	38.0
Oromo	103	26.8
Gurage	53	13.8
Others^c^	38	9.9
Family size		
1–2	55	14.3
3–4	149	38.8
≥5	175	45.6
Mean ± SD	4.6 ± 2.2	
Monthly income ($US)		
<25	51	20.9
25–50	133	54.5
>50	60	24.6
Median	37.5	
Education		
No education	226	58.9
Primary	67	17.4
Secondary	52	13.5
Above secondary	39	10.2

^a^Standard deviation

^b^Catholic, Jehovah Witness and traditional religion

^c^Welayta, Somali and Afar

Of total respondents, around 239 (62.2 %) had previous knowledge of cancer PC services. Out of this, 86.6 % (207 out of 239) respondents were ≥35 years of age and knowledgeable of PC services. About 113 (47.3 %) respondents had knowledge of PC but they had no any formal education. The majority 85 (60.7 %) respondents reported that they had no knowledge of PC services for cancer (Table 2).

**Table 2:** Percent distribution of respondents by their previous knowledge of PC services and selected socio-demographic characteristics, Tikur Anbesa Specialized Hospital, Addis Ababa, Ethiopia, 2014

Characteristics	Knowledge^c^	
	Yes, N = 239 (100 %)	No, N = 145 (100 %)
Age (years)		
<35	32 (13.4)	9 (6.2)
≥35	207 (86.6)	136 (93.8)
Educational status		
No education	113 (47.3)	113 (77.9)
Primary	50 (20.9)	17 (11.7)
Secondary	40 (16.7)	12 (8.3)
University/college	36 (15.1)	3 (2.1)
Family size		
1–2	33 (13.8)	22 (15.7)
3–4	116 (48.5)	33 (23.6)
≥5	90 (37.7)	85 (60.7)
Missing	0	5 (3.4)
Income ($US)		
<25	43 (18.0)	8 (9.5)
25–50	68 (28.5)	65 (77.4)
>50	49 (20.5)	11 (13.1)
Missing	79 (33.1)	61 (42.1)
Occupation		
Farmer	34 (14.2)	52 (35.9)
House wife	82 (34.3)	57 (39.3)
Employed^a^	93 (38.9)	15 (10.3)
Merchant	7 (2.9)	10 (6.9)
Others^b^	23 (9.6)	11 (7.6)

^a^Government, private or NGO

^b^Others—daily laborer, unemployed, disabled, retired, and student

^c^Previous knowledge before this interview

Accessibility of PC services by client perspective includes: “highly accessible Counseling Service in the hospital” was 6 % of the respondents whereas around 15.4 % of client’s reported the services totally not accessible in the hospital or the client doesn’t know the existence. No respondent (0 %) reported high accessibility of receiving 24 h telephone support and Cancer advisory services in the hospital. As summarized by Table 3, another major level of responses that client reported “highly accessible” were Performance of home activities (12.0 %). But, at this level, comparatively a minimum number of clients (3.9 %) had responded as home activities were not applicable in the hospital (Table 3).

**Table 3:** Percent distribution of respondents with cancer by level of response and selected accessibility characteristics, Tikur Anbesa Specialized Hospital, Addis Ababa, 2014

Service characteristics	Level of response^a^ and client satisfaction^a^					
	Not applicable^b^, n (%)	Fully satisfied, n (%)	Low^c^, n (%)	Moderate^d^, n (%)	High^e^, n (%)	Total n (%)
Counseling services	59 (15.4)	171 (44.5)	90 (23.4)	41 (10.7)	23 (6.0)	384 (100)
Service brochure and benefit	210 (54.7)	21 (5.5)	135 (35.2)	17 (4.4)	1 (0.3)	384 (100)
Books and videos library	236 (61.5)	15 (3.9)	112 (29.2)	16 (4.2)	5 (1.3)	384 (100)
Relaxation class	179 (46.6)	30 (7.8)	151 (39.3)	19 (4.9)	5 (1.3)	384 (100)
Drop counseling and support service	49 (12.8)	164 (42.7)	110 (28.6)	42 (10.9)	19 (4.9)	384 (100)
24 h telephone support and cancer advisory	228 (59.4)	31 (8.1)	117 (30.5)	8 (2.1)	0	384 (100)
Home nursing service	162 (42.2)	35 (9.1)	54 (14.1)	105 (27.3)	28 (7.3)	384 (100)
Perform home activities	15 (3.9)	30 (7.8)	97 (25.3)	196 (51.0)	46 (12.0)	384 (100)
Monitory allowances	263 (68.5)	17 (4.4)	98 (25.5)	6 (1.6)	0	384 (100)

^a^By client perspective

^b^Services doesn’t exist/client don’t know

^c^Low accessibility

^d^Moderate accessibility

^e^High accessibility

Up on interview on use of PC services in the last 12 months cancer diagnosis, about 69 % (majority) reported they had PC services from TASH. About 25.5 % (minor) patients responded as they had Community Based Cancer Support group services (Table 4).

**Table 4:** percent distribution of clients with cancer by selected basic palliative care services they had in the last 12 months, Tikur Anbesa Specialized Hospital, Addis Ababa, 2014

Services used	Yes (n)	Percent
Had services of cancer information and support	120	31.3
Had community based cancer support group services	98	25.5
Had face to face counseling services	172	44.8
Had any of palliative service from its center (TASH) in the last 12 months	265	69.0

Percentage total is more than 100 because there is multiple response

The multivariable logistic regression model carried out using binary analysis observed six variables, such as: client’s previous knowledge, physical well being, social well being, monthly income and marital status were significantly associated with respondent’s utilization of PC services. This utilization was considered for client who had above average response to selected basic utilization category. Respondents who had previous Knowledge of PC services to cancer were 26.9 times (AOR = 26.9, 95 % CI 12.3, 59) more likely to use the services as compared to those who were not knowledgeable. Clients who had little physical well being were 3.1 times (AOR = 3.1, 95 % CI 1.96, 4.9) more likely to use PC services than those who had no physical well being, some physical well being, and/or full physical well being. Respondents who had full social well being were 1.7 times (AOR = 1.7, 95 % CI 1.01, 2.8) more likely to use PC services than those who were lacking social services, had little social services, or some social services. Respondents who had $US 25–50 monthly income were 0.25 times (AOR = 0.25, 95 % CI 0.09,0.7) less likely to use PC for cancer than those who had below $US 25 monthly income per respondent. And, respondents who had single Marital status at data collection period were 55.4 times (AOR = 55.4, 95 % CI 1.2, 2660.4) more likely to use PC services for cancer than those who were divorced. Respondents who had some physical well being (50 % response were “yes”) had association with utilization of PC than those who had no, little, or full physical well being in binary regression, but not significantly associated by adjusted odds ratio (Table 5).

**Table 5:** Multivariable logistic regression model for client’s use of palliative services by selected characteristics, Tikur Anbesa Specialized Hospital, Addis Ababa, Ethiopia, 2014

Characteristics	Utilized PC							
	Yes, n = 210 (54.7 %)	No, n = 174 (45.3 %)	COR	95 % CI		AOR	95 % CI	
				Lower	Upper		Lower	Upper
Knowledge								
Knowledgeable^b^	180 (85.7)	59 (33.9)	11.7**	7.1	19.2	26.9**	12.3	59.0
Not knowledgeable	30 (14.3)	115 (66.1)	1.0+			1.0+		
Little physical well being^c^								
Yes	158 (75.2)	83 (47.7)	3.3**	2.2	5.1	3.1**	1.96	4.9
No	52 (24.8)	91 (52.3)	1.0+			1.0+		
No physical well being^d^								
Yes	123 (58.6)	126 (72.4)	1.9*	1.2	2.9	0.8	0.5	1.3
No	87 (41.4)	48 (27.6)	1.0+			1.0+		
Social/family well being^e^								
Yes	171 (81.4)	120 (69)	2**	1.2	3.2	1.7**	1.01	2.8
No	39 (18.6)	54 (31)	1.0+			1.0+		
Monthly income^a^ ($US)								
<25	41 (27 %)	10 (10.9)	1.0+			1.0+		
25–50	70 (46.1 %)	63 (68.5)	0.27**	0.13	0.59	0.25**	0.09	0.7
>50	41 (27 %)	19 (20.7)	0.53	0.22	1.27	0.3	0.1	1.01
Marital status								
Single^b^	7 (3.3)	1 (.6)	42.0**	2.1	825.7	55.4**	1.2	2660.4
Married	157 (74.8)	81 (46.6)	11.6*	1.4	98.2	7.1	0.47	109.6
Widowed	38 (18.1)	47 (27)	4.8	0.6	42.0	1.4	0.08	22.9
Separated	7 (3.3)	39 (22.4)	1.1	0.1	10.4	0.39	0.02	7.0
Divorced	1 (0.5)	6 (3.4)	1.0+			1.0+		

* Significant by binary analysis

** Statistically significant at (p < 0.05)

^+^Reference category

^a^Missed case, 58 (27.6 %)

^b^Large confidence interval

^c^More than 75 % of physical problems list responded

^d^All physical problems in the list responded as “exist”

^e^All social relationship problems responded as “good” by respondents

## Discussion

This Institution based cross-sectional study attempted to assess knowledge, accessibility and utilization of PC services among adult cancer patients in TASH, Addis Ababa, Ethiopia. In this study, majority respondents (62.2 %) were previously knowledgeable about PC services to cancer. Among this, more than 4 out of 5 respondents (86.6 %) were older aged (207 out of 239), greater than 35 years of age. This finding was in line with African, USA (86.6 % vs 48.9 % and 83 % respectively) and other studies [25, 28, 32]. This may be due to age factor as one of the risk factor for the episode of cancer as compared to those who were younger.

Out of the total respondent with previous knowledge of PC services, about 9 out of 10 (92.1 %) patients (220 out of 239) reported accessibility of PC services by their perspective at TASH. This was even higher than findings in the abroad [25]. The difference may be due to different cut-off points used in either of the study. In this study, the cut-off point used was the median value of responses.

Among respondents who had previous knowledge of PC services asked for its utilization in the last 12 months of cancer experiences, majority reported (206 out of 239) that they had at least one PC service category from its center at TASH in the last 12 months of data collection period. This was higher from other study findings outside Ethiopia (86.2 % vs 66.3 % and 66 %) [24, 25]. This difference may be due to consideration of percentage for whole or more service categories in other studies. In this study, any one of palliative services given to the client was considered in the percentage of services used.

This study showed that respondents who had used PC services to cancer were statistically significantly associated (AOR = 26.9, 95 % CI 12.3, 59) with those who had previous knowledge of the services. This relation is similar with the fact that lack of public awareness about early detection, treatment of invasive cancer and PC services as a barrier in countries with limited cancer treatment services, like Ethiopia [18].

PC utilization were statistically significantly associated (AOR = 3.1, 95 % CI 1.96, 4.9) with Clients who had little physical well being. No other studies were found on relationship of this regard. This association may show that the need for QOL were higher among those with little physical well being as compared to no physical well being or full physical well being. This was because those who had full physical well being were not illed aggressively to fear for death or need quality living as compared to those with little physical well being for use of PC services. Those respondents with no physical well being were seemed to be lost the expectations for survival. Due to this, they had little use of PC services comparatively.

PC utilization were statistically significantly associated (AOR = 1.7, 95 % CI 1.01, 2.8) with respondents who had full social well being (no any of social problems encountered). This may be due to absent discrimination motivated the respondent to use PC services and become not to be fearful for exposure and confidentiality as compared to those respondents who had at least some social well being. Comparative study was not available.

PC utilization were statistically significantly associated (AOR = 0.25, 95 % CI 0.09, 0.7) with respondents who had $US 25–50 monthly income. This was contradictory with the findings in New York and India [26, 27, 33] in that high income respondents were more likely to use palliative services than low income. This difference probably indicates that those respondents who were poor or low household monthly income in this study likely to be more fearful of death or disability as compared to those who had relatively higher family monthly income. This fear may be related to fear of losing their child care taker after their disability or death. This is because they have minimum income to accommodate all the child care process in the future.

PC utilization were statistically significantly associated (AOR = 55.4, 95 % CI 1.2, 2660.4) with respondents who have single marital status. The Kuwait study was contrary to this association [28]. This difference may be due to high need of QOL for single respondents as compared to those who had at least first marriage before and now divorced. This may be because of respondent’s need to be kept healthy for getting marriage in the future as not had experienced so far.

## Conclusion

More than just an average respondents being treated on TASH had knowledge of cancer PC services. High number of respondents (9 out of 10) reported problems on accessibility of PC services for cancer in TASH. Respondents previous knowledge of pc services; little physical well being; social well being; monthly family income; and marital status were statistically significantly associated with respondent’s utilization of cancer PC services at TASH. Health services providers at TASH will be recommended to have sustainable health education program on cancer PC services to the victims. Facilitations required for the clients have to be made easily accessible based on the standards mentioned in this Hospital. Community Mobilization through the media, posters and face to face communication on Community Health Day (CHD) focusing on PC services to cancer must be addressed by Addis Ababa town administrations and policy makers. The Federal Democratic Republic of Ethiopia, MOH must plan to build additional center in Addis or elsewhere in the country for more awareness creation to the clients and achievement of cancer PC services by the majority.

## Abbreviations

MOH: ministry of health
PC: palliative care
QOL: quality of life
TASH: Tikur Anbesa Specialized Hospital
